# Editorial Chit-Chat

**Published:** 1873-01

**Authors:** 


### EDITORIAL CHIT-CHAT.

PROFITABLE SUTLING.—Opium selling in the Sandwich Islands must be profitable. The privilege of selling opium for one year, was recently bid off at auction for twenty-one thousand dollars.

THE ENVELOPE.—Look out for the envelope in this number. Enclose your subscription in it, fill up the blank, and mail it to us. We will credit you with it in our next issue. Those who are more than a year in arrears, will find a suitable blank for them to fill.

AT IT AGAIN.—Our old friend, Cal. W. Gutelius, Esq., has commenced the publication of a very neat looking weekly—the *Public Press*, at Northumberland, Pa. Cal. gives the Northumberland people a journal that is an honor to the place. It should be liberally supported.

A NEW VEIN.—Our friend and correspondent, "Ned," has assumed a new *role* in this number of the BISTOURY, that of Moralist.—Those who have heretofore enjoyed his spicy and droll communications, will scarcely recognize him.

MUSICAL INSURANCE.—Our musical friend, having an elegant piano which he desired insured, asked James, that sly jester of the board of underwriters, what he would do in case the piano took fire. "Play on it," replied James. The policy was immediately canceled.

WARNING.—The young lady in New Haven, Conn., who addressed a letter to a "Dr. Crandall," care of the Eye and Ear Institute, in this city, is informed that the so-called "doctor" is an impostor, and that she had better send her photographs to parties with whom she is better acquainted.

EXASPERATING.—We have just been consulted by a young gentleman who declares himself to be upon the brink of insanity, by reason of being again compelled to read a piece of doggerel, which has sought to entice him to a toy shop, for the last dozen years or so. He earnestly calls for a new deal.

COON CHASE.—Mr. Silkman, of Scranton, Pa., recently undertook to capture a coon, which had injured his wife, and chased the animal into the *Sunday Free Press* printing office, where cases of type and other materials were upset and the office completely demolished.

SOUP FOUNTAINS.—Paris is a queer place.—Should be peopled with Yankees, and one would suppose it is, to read of the odd things they occasionally do there. Their last novelty is a soup fountain, where the poor can go and fill their hungry stomachs with delicious soup, free of charge.

SOLID.—Henry W. Strang informs us that the disposition of purchasers of silver ware this season is to buy it *solid*. He has sold an immense amount of solid silver ware, and very little plated. Those in want of beautiful gifts for the holidays, will find a choice variety at Strang's.

HALLS.—The magnificent store of Hall Bros. is looking more elegant than ever before, if possible, in its holiday attire. It is thronged with busy buyers, all day long, selecting the usual gifts of the season for friends and sweethearts.

RIVAL OF TEA.—A new beverage has been recently discovered and promises to be a rival of our tea. It is the fruit of a Brazilian tree known as *guaraca*, the active principle of which is an alkaloid, identical with that found in tea and coffee. It is said to be a specific for sick headache, and is being sold by our druggists, in the form of a powder, for that purpose.

LARGE SALARY.—There exists a professional gentleman, in this city, who has been offered twenty-five thousand dollars for one year's services, in the interest of other parties. We know this to be a fact. That exceeds the offer which it is asserted Hon. Schuyler Colfax received to become the editor of the *Tribune*, and equals the salary of the President of the United States.

CRIPPLE MANUFACTORY.—This strikes one as a singular industry, yet we are assured by an English journal, that the police of London have just discovered an institution where children are taken and their limbs deformed, so that they may become objects of charity and successful beggars. A regular tariff of prices is demanded by these contemptible surgeons. A bandy-leg, two pounds, while for a complete maiming, four pounds is charged. The managers of the concern, and a dozen or so patients, have been imprisoned.

---

TOO THIN.—Mr. John J. Fulton, Counselor-at-Law, Attorney for Divorces, etc., is respectfully informed that we expect and demand payment in advance for cards from such as he. Divorce Lawyers have not been very profitable advertisers for journals in this country. We would rather have these divorce dealers "pay in advance," the same terms they offer their clients. It is safer.

---

A BLESSING.—We hear of an old chap "out west," who was sentenced to be hung by a vigilance committee, for an unimportant crime,—horsestealing, murder, or something of that sort; but the executioners had to give up the job; they couldn't hang the fellow successfully, or at least to their own satisfaction, by reason of an immense wen growing upon the throat of the victim. The culprit is cultivating that tumor as a blessing in disguise.

---

ELEGANT.—W.·. Brother C. W. Shipman, of this city, was recently surprised with a magnificent solid silver service, costing one thousand dollars. It was given him by the members of Ivy Lodge, No. 397, F.·. A.·. M.·., in testimony of their appreciation of the manner in which he has served that body, as Master, for the past three years. He has since been unanimously re-elected to fill the position for a fourth term, a distinction that has never been accorded any other Master in that Society.

---

MACHINE FOR PREVENTING SEA-SICKNESS.—Mr. Henry Bessemer, the steel manufacturer, has just devised an apparatus for preventing sea-sickness. It consists of a large, circular saloon, on axles, with machinery designed to overcome movements in all directions, from oscillation, either from the waves or the motion of the ship, so that the floor shall be kept horizontal under all conditions. Eminent engineers, who have examined the model, say it will accomplish the object desired.

---

ACADEMY OF SCIENCES.—This scientific body are preparing for another meeting soon, at which the following papers will be read: The Ralston Coal Region, by Col. H. M. Smith; Architecture, as related to Great Fires, by Rev. T. K. Beecher; Bearing of recent Ocean Dredging on Geological Theories, by Prof. Ford; Compressed Air as a Mechanical Motor, by Mr. G. W. Farnham; Testing Ores of Iron, Laboratory Researches, by Dr. W. H. Gregg; Recent Discoveries in Optics, as applied to the eye, by Dr. T. S. Up de Graff.

---

VERY DANGEROUS.—There should be a flagman stationed at the R. R. crossing at South Water street. It has grown to be a great thoroughfare, and we have recently witnessed some very narrow escapes from accident at this point The great number of trains passing upon the double tracks, each way, with no one to signal their coming, renders it one of the most dangerous crossings in our city.

---

USE FOR OLD BOOTS.—A distinguished German professor has discovered a process by which tannin can be removed from leather, restoring it to the condition it was before tanning. A firm in Paris are even now engaged in making a very excellent quality of glue from old boots and shoes which are gathered up about the city, and have so found a market value. We doubt not a very good "gelatine" can be made from the same article, so that the delicious article of food known as "wiggle," can be afforded at a much cheaper rate than now.

---

LECTURES.—It is amazing to see with what slender claims for a hearing, many of the so-called lecturers thrust themselves before the public. The most recent candidate for Lyceum honors, is Dr. Paul Shœppe, who was for four years under sentence of death for the alleged poisoning of Miss Steinnick, at Carlisle, Pa.—It seems the Doctor relies upon the notoriety given him through his imprisonment, sentence and acquittal, to give him sufficient audiences to make his lecture tour profitable. He opened in Philadelphia, in Assembly Building, to a very small audience, a few days ago.

---

SIMPLE AND RELIABLE.—It is usually the case that the simplest inventions are the best. This assertion is supported in the fact of a simple discovery for a positive sign of death. The French Academy of Sciences offered twenty thousand francs for such a test, one that could be applied at any time by non-medical persons, requiring no apparatus, and unmistakable in its indications. A large number have been proposed, but the latest, and the one most likely to win, is offered by Dr. Hugo Magnus, of Breslau. In consists simply in *tying a string around the finger of the person supposed to be dead.* If there is the least spark of life left, the whole finger, from string to tip, will gradually turn a bluish red, from the engorgement of the veins. This will occur, no matter how profound the coma or trance, and will not be present in death.

---

NONE, IF YOU PLEASE.—The *Food Journal* says that three men have been arrested in Paris for manufacturing sausage of anything but healthy, or one would suppose, palatable materials. A very bad smell, emanating from the wholesale sausage establishment, induced some of the most extensive buyers to inspect the concern. They discovered that ye gentle sausage man was manufacturing a very desirable article from the bodies of buried communists, and the flesh of dogs and cats, collected by the "chiffonniers" of Paris. Strange to say, the concern was suppressed.

---

ARTIFICIAL EYES.—A French journal gives a detailed account of the manufacture of artificial eyes in Paris, from which the curious fact appears that the average sale per week, of eyes intended for the human head, amounts to four hundred. One of the leading dealers in this article carries on his business in a saloon of great magnificence; keeping a servant with but one eye, so that the effect of any eye desired by a customer is conveniently tried in this servant's head, enabling him to judge very readily as to the appearance it will produce in his own head.

We know a dealer in this country, who has driven a successful business in artificial eyes, by reason of his being able to exhibit the effect of his goods upon the appearance of his wife.—She has but one natural eye—the other being artificial and beautifully matched.

---

A HORSE DEVICE.—A chap called upon us today, with an ingenious apparatus for stopping runaway horses. It consists of a pair of nose-stoppers attached to a bit, which close over the nostrils, stopping the animal's "wind," when a cord is pulled for the purpose. At the same pulling of the cord a "blinder" is thrown over the eyes, so that the horse is completely blindfolded, and as this condition is very unfavorable to running, why, he stops. He wanted to try the concern on our ponies, but when we informed him that they had reached years of discretion—about 80—and were not given to running—*not much*, and that we would prefer some diabolical apparatus to *make* them run, he thought perhaps the concern would have no intrinsic value in our hands; we thought so too, and therefore spared the ponies the disgrace of appearing in public in such a humiliating dress.

---

COST OF GAS.—We have read one or two communications in the *Advertiser*, relative to the cost of gas in this city, which has induced us to investigate the matter, and from which we learn the following facts:

The cost of gas, to the consumer, in New York, Boston and Rochester, is $3.00 per thousand cubic feet. In Cincinnati, $2.50. The *Gas Consumers' Guide*, most excellent authority upon the subject, says that the actual cost of production, with coal and labor at present prices, cannot exceed, if any, $1.50 per thousand cubic feet. Add to the first cost, in justice to the gas companies, the leakage and loss from bad customers, the cost of production would not exceed $1.75 per thousand cubic feet, leaving a good, *liberal* margin of profit to the Company that charges $4.00 a thousand cubic feet for their gas.

This estimate is based upon the theory that one ton of coal will make 9,000 feet of pure gas, about one chaldron of coke and about ten gallons of tar. If the coal is good, such as is used in this city, it will do better than our estimate. Under such circumstances, we deem it not unreasonable to assert that $4.00 is entirely too much to pay for gas, and that the Company who have a monopoly of furnishing it at such a figure, have stock that pays an enormous dividend, and which should be reduced by competition.

---

SPELLING.—A lively time was had among the scholars of public school No. 3, a few days ago. A valuable prize was offered to the best speller in the school, and in order to determine who that individual was, one hundred words were pronounced in their hearing, which were written upon sheets of paper by those contending for the prize. About two hundred pupils entered the list, most of whom did very creditable spelling, much better, we imagine than the same number of successful business men could have done. MASTER JAMES FINLAY, of C Seniors, carried off the prize, having spelled eighty-three words of the hundred, correctly.—We give the list below. Have some one pronounce them for you and see whether you can do as well as Master Finlay.

Geo. Osborne, of A Seniors, spelled 82 of the words; Miss Stella Farnham, A Seniors, and Joseph Eustace, of B Seniors, each spelled 81 correctly; Master F. E. Upkike mastered 79.—It will be seen from the list of words given, that they did surprisingly well. We doubt whether there is a school in the city that can exhibit as many good spellers as No. 3. They are a credit to Mr. Coburn and his assistants, and exhibits the fact that the teachers bestow extraordinary care in teaching proficiency in spelling,—the very foundation of an English education. Here are the words:

| | |
|---|---|
| 1. Educational. | 51. Pieces. |
| 2. Gymnasium. | 52. Cartilage. |
| 3. Ellipsis. | 53. Negotiable. |
| 4. Spectacles. | 54. Paroxysm. |
| 5. Victuals. | 55. Parquet. |
| 6. Summons. | 56. Parliament. |
| 7. Politics. | 57. Pamphlet. |
| 8. Composing. | 58. Opodeldoc. |
| 9. Individuals. | 59. Omniscience. |
| 10. Conscience. | 60. Teetotaler. |
| 11. Reciprocal. | 61. Erysipelas. |
| 12. Interrogative. | 62. Ecstasy. |
| 13. Conjugation. | 63. Extravagance. |
| 14. Synopsis. | 64. Diphtheria. |
| 15. Auxiliary. | 65. Epitaph. |
| 16. Arrangement. | 66. Alpaca. |
| 17. Participle. | 67. Vill. |
| 18. Transitive. | 68. Epizootic. |
| 19. Sarsaparilla. | 69. Caraway. |
| 20. Corresponding. | 70. Separation. |
| 21. Politest. | 71. Traveler. |
| 22. Foregoing. | 72. Satellite. |
| 23. Mucilage. | 73. Eyelet. |
| 24. Mien. | 74. Equinoctial. |
| 25. Pony. | 75. Chagrin. |
| 26. Peddler. | 76. Champagne. |
| 27. Robbed. | 77. Villainous. |
| 28. Baccalaureate. | 78. Brilliancy. |
| 29. Amateur. | 79. Characteristic |
| 30. Chameleon. | 80. Phæton. |
| 31. Chamois. | 81. Tureen. |
| 32. Weird. | 82. Turbot. |
| 33. Homeopathic. | 83. Turquois. |
| 34. Therapeutic. | 84. Phalanx. |
| 35. License. | 85. Pharisee. |
| 36. Hieroglyphic. | 86. Cylinder. |
| 37. Rhapsody. | 87. Polygon. |
| 38. Rhomboid. | 88. Arithmetical. |
| 39. Rhythm. | 39. Shock-full. |
| 40. Riddance. | 90. Descending. |
| 41. Ridiculous. | 91. Criss-cross. |
| 42. Rubescence. | 92. Criticiser. |
| 43. Ruffed. | 93. Elasticity. |
| 44. Scour. | 94. Magnesia. |
| 45. Slurred. | 95. Quinsy. |
| 46. Teuton. | 96. Requiem. |
| 47. Pseudonym. | 97. Assassin. |
| 48. Hazel. | 98. Linament. |
| 49. Douche. | 99. Obeisance. |
| 50. Similarity. | 100. Coverlet. |

---

COCKROACHES.—Ever cultivate any? They are prolific,—very. No trouble to raise them; just supply plenty of hot-water pipes in your house, borrow one little roach from your neighbor, and we will guarantee you a million or so, as a result of your enterprise, within thirty days. It is so jolly to see them perching upon a nice, cold turkey, in your pantry,—it gives it a sort of animated appearance, and robs death of its hideousness, you know. Why, you would never dream you were lunching upon *dead* turkey,—the very idea is repulsive; but contrariwise, you will find it to be a very lively and animated bird. Then, when you open your receptacle for cold victuals, it is endless amusement to see the little rascals run. If you can chase one down the hollow stem of a champagne glass, he will generally stick there, and by placing a dried currant upon his hind legs, he will amuse you by the hour in tossing the thing in the air, equal to any circus performer. He can creep through the closest fitting sugarbowl-cover, swim across a wider pan of milk, and hide more successfully in your soup, than any little cuss of equal dimensions you ever saw.—We have tried them in all their varied performances, and pronounce them to be the most versatile little wretches within our knowledge of animated nature. But wife became tired of them,—thought the amusement was monotonous, as it were; and, urged on and abetted by cook, and other members of the household, commenced an indiscriminate slaughter of them. She first ordered them bathed in hot water,—it made them dance around rather lively, but they were always on hand next day, as jolly as ever. Next, she had us on our knees, squirting carbolic acid into every crack and crevice—their humble abiding-places. This process, we are sorry to say, crippled many of them, so that an unusual number failed to creep from our morning hash, when they were summoned so to do by the cook and the frying-pan. We ordered the abandonment of this experiment, as it imparted a very peculiar flavor to the hash. We think there is something about the mixture of cockroaches and carbolic acid that is akin to succotash—we are sure we don't like it. Fumigating the kitchen and closets with sulphur was a tolerably successful experiment, for the sulphur coating upon the backs of the roaches, we thought improved their appearance, without doing them any perceptible injury. Finally, an artful dodger, Palmer by name, a foundryman, who resides in Brooklyn, we believe, persuaded our better-half to sprinkle *borax*—powdered, he said—in the cracks and crevices where our little innocents

—the roaches—dwelt. We did it, thinking no harm would come of such a simple performance, and to this day we have not been able to interview even one of them. We have been searching, every blessed day, for our old friends, the roaches, but they have gone—gone we know not whither—and seemingly without a regret. Since then our meals have lost their accustomed flavor, our gymastic performances have ceased, and we stand amazed at the consequences of that borax.

